# High CXCR4 Expression Predicts a Poor Prognosis in Resected Lung Adenosquamous Carcinoma

**DOI:** 10.7150/jca.36498

**Published:** 2020-01-01

**Authors:** Qiaoliang Zhu, Rongkui Luo, Jie Gu, Yingyong Hou, Zongwei Chen, Fengkai Xu, Lin Wang, Wei Mao, Chunlai Lu, Di Ge

**Affiliations:** 1 Department of Thoracic Surgery, Zhongshan Hospital, Fudan University, Shanghai, P. R. China.; 2 Department of Pathology, Zhongshan Hospital, Fudan University, Shanghai, P. R. China.; 3 Shanghai Respiratory Research Institute, Zhongshan Hospital, Fudan University, Shanghai, P.R. China.

**Keywords:** CXCR4, lung adenosquamous carcinoma, prognosis

## Abstract

**Background:** Primary adenosquamous carcinoma (ASC) is a rare malignant tumor in the lung and its biological behavior has not yet been thoroughly described. In this study, we aimed to explore the clinical and biological role of CXCR4 in patients with resected lung ASC.

**Methods:** We retrospectively reviewed the clinical records of patients with histologically confirmed lung ASC who underwent surgical resection with systematic lymph node dissection. Immunohistochemical staining was performed to detect the expression of CXCR4 in tumor tissues. The correlation between CXCR4 expression and clinicopathological characteristics were evaluated. The association between CXCR4 expression and patients' prognosis was analyzed by Kaplan-Meier and Cox regression. Moreover, we performed *in vitro* studies including CCK8, transwell and cell apoptosis to explore the potential role of CXCR4 in lung ASC.

**Results:** A total of 78 patients with resected lung ASC were reviewed. Seventy (89.7%) patient tumors expressed CXCR4, with high level of CXCR4 expression observed in 45 (57.7%) cases. *In vitro*, CXCR4 conferred no difference in proliferative capacity but increased invasive potential, enhanced chemoresistance and inhibited apoptosis of lung ASC. Clinically, high CXCR4 expression was significantly associated with solid ASC, lymph node metastasis and advanced TNM stage. Patients with high CXCR4 expression and solid ASC had decreased disease-free survival and overall survival.**Conclusions:** CXCR4 was commonly expressed in lung ASC tumors. High CXCR4 expression might be a novel marker in predicting a poor prognosis in resected lung ASC and might serve as a potential therapeutic target.

## Introduction

Lung cancer is the leading cause of cancer deaths worldwide^1^. The non-small-cell lung cancer (NSCLC) is the most frequent type of lung tumors in humans, with two major histological subtypes: adenocarcinomas (AC) and squamous cell carcinomas (SCC). Adenosquamous carcinoma (ASC) is a relatively rare histology type of NSCLC, accounting for 0.4% to 4.0% of lung cancers^2^. According to World Health Organization classification, ASC is defined as a carcinoma showing the components of both AC and SCC in at least 10% of the tumor^3^. But this composite tumor is not simply a malignancy with mixed components, it presents more aggressive characteristics than “pure” AC and SCC, with rapid growth, high invasiveness, early metastasis, and a very poor prognosis^4^, ^5^.

C-X-C chemokine receptor 4 (CXCR4) is a 352 amino acid rhodopsin-like G protein-coupled receptor (GPCR) expressed in multiple cell types, which selectively binds to its sole ligand-the CXCL12 also known as stromal cell-derived factor 1 (SDF-1). The binding of CXCL12 to CXCR4 induces intracellular signaling through several divergent pathways, playing an important role in cell survival, proliferation, migration, and metastasis of several cancers, including breast^6^, ovarian^7^, colorectal^8^, and pancreatic carcinomas^9^, among others. It has also been shown that many NSCLC cell lines express high levels of CXCR4 and that SDF-1-actialvated CXCR4 promotes migration and invasion of these cell lines *in vitro*^10^. Whereas neutralization of the CXCR4/SDF-1 axis is associated with a decrease in NSCLC metastasis* in vivo*^11^.

Several retrospective studies had reported the association between CXCR4 expression and clinical outcome in NSCLC^12^-^14^, suggesting that the CXCR4/SDF-1 axis plays an important yet incompletely defined clinical role in NSCLC. Since NSCLC is a group of malignancies with extensive heterogeneity, outcomes might be disparate according to different subtypes. To the best of our knowledge, no studies regarding the role of CXCR4 in lung ASC have been performed. On this background, we currently aimed to detect CXCR4 expression in patients with resected lung ASC. In addition, we explored the potential correlations between CXCR4 expression and clinicopathological parameters and determined the prognostic value of CXCR4 in resected lung ASC. Besides, we performed some functional experiments *in vitro* to further verify the biological role of CXCR4 in human ASC cell line.

## Materials and Methods

### Specimen collection and tissue samples

From June 2014 to June 2018, 78 patients underwent complete tumor resection and lymphadenectomy were included in this study. All sections of paraffin-embedded primary tumor tissues and lymph nodes were reviewed by 2 experienced pathologists with no knowledge of the patients' information or the purpose of the research. Histologic diagnosis of ASC was established when both squamous and glandular components of the tumor were more than 10% of the tumor. As to proportion of glandular and squamous components, ASC were subdivided into 3 groups, according to the criteria proposed by Gawrychowski 4: an adenocarcinoma (ACC)-predominant (60-90%) group, a balanced (40-60%) group, and a squamous cell carcinoma (SCC)-predominant (60-90%) group. Histological subtypes of glandular component were determined by the new IASLC/ATS/ERS multidisciplinary classification^15^. Tumors were classified into non-solid ASC when glandular component showing acinar, lepidic, micropapillary, or papillary growth pattern, otherwise classified into solid ASC. This retrospective study was approved by the ethics committee on human research of Zhongshan Hospital, Fudan University, Shanghai, China. Written informed consent was obtained from all patients.

### Immunohistochemical staining and evaluation

Immunohistochemical staining was performed as described by Lu and his colleagues^16^. Sections were immunostained with the primary antibodies CXCR4 (Clone 44716, R&D Systems, Minneapolis, MN, USA), detected with the EnVision procedure (DAKO). According to staining intensity and percentage of positive tumor cells, the final staining score was given: 0 (negative); 1 (<50% weak or strong positive cells); 2 (>50% weak positive cells); 3 (>50% strong positive cells). For statistical analysis, score (0, 1) and (2, 3) were considered as low and high expression, respectively.

### EGFR mutation analysis

Genomic DNA was isolated and purified from formalin-fixed, paraffin-embedded blocks with adequate tumor tissue using a DNeasy Tissue Kit according to the manufacturer's instructions. Then, we used PCR-single-strand conformational polymorphism analysis to detect mutations in exons 18, 19, 20, and 21 of the EGFR gene as previously described^17^.

### Clinicopathological evaluation

Medical records were reviewed to extract data on clinicopathologic characteristics, including age at diagnosis, gender, smoking history, subtype of ASC, predominant subtype of AC, presence of micropapillary, tumor differentiation, visceral pleural invasion, lymphovascular invasion, tumor size, T stage, lymph node metastasis, pathologic tumor-node-metastasis (TNM) stage, mutational status of EGFR (exons18-21), and CXCR4 expression. TNM stages were evaluated in accordance with the 7th edition of the lung cancer staging classification system^18^.

### Follow-up

After surgery, patients were followed up every 3 months during the first year and every 6 months thereafter. Overall survival (OS) was defined as the time elapsed from the date of surgery to death or the last follow-up visit for censored patients. Disease-free survival (DFS) was defined as the time elapsed from the date of surgery to local relapse or distant metastasis. Complete information on patient survival was available for 74 patients, and 4 patients were lost to follow-up. The last date of follow-up was December 31, 2018.

### Cell lines culture and transfection

The human ASC cell line H596 was purchased from the Chinese Academy of Sciences. The cells were cultured in DMEM, supplemented with 10% fetal bovine serum and 100 IU/ml penicillin/streptomycin in a humidified incubator, under 95% air and 5% CO2 at 37℃. Lipofectamine 2000 (Invitrogen, Carlsbad, USA) was applied for transient transfection. Predesigned siRNA duplexes were purchased from Sangon Company. The sequences of siRNA-CXCR4-1 are 5'-GGUACUUUGGGAACUUCCU-3' (F) and 5'-AGGAAGUUCCCAAAGUACC-3' (R). The sequences of siRNA-CXCR4-2 are 5'-CCUGUCCUGCUAUUGCAUU-3' (F) and 5'- AAUGCAAUAGCAGGACAGG-3' (R). The sequences of siRNA-CXCR4-3 are 5'-GUGAGUUUGAGAACACUGU-3' (F) and 5'-ACAGUGUUCUCAAACUCAC-3' (R). The normal control group was cells transfected with a non-targeted scramble sequence of 5'-UUCUCCGAACGUGUCACGU-3' (F) and 5'- ACGUGACACGUUCGGAGAA-3'(R). The knock-down group was cells transfected with siRNA-CXCR4 sequences. The transfection procedure was performed as previously described^19^.

### qRT-PCR analysis and Western blot assay

The methods of total RNA extraction and qRT-PCR analysis were consistent to the previous research^19^. The primers of CXCR4 are 5'- TGTCATCTACACAGTCAACCTC-3' (F) and 5'- CAACATAGACCACCTTTTCAGC-3'(R). The primers of β-actin are 5'-TGACGTGGACATCCGCAAAG-3' (F) and 5'-CTGGAAGGTGGACAGCGAGG-3' (R). Western blot analysis was carried out as described previously^19^. Anti-CXCR4 antibody (ab181020) was purchased from Abcam Corp. (Cambridge, UK).

### CCK-8 assay, Transwell assay and cell Apoptosis analysis

CCK-8 assay was performed as previously described^20^. The OD values were detected at 24, 48, 72, 96 hours after transfection, respectively. To analyze whether CXCR4 expression has any impact on the response to paclitaxel (PTX) treatment, cells were treated with increased concentration of PTX for 72 hours after transfection.

Transwell assay was also performed as previously described^20^. Briefly, cells (1×10^5^) were suspended in 200μl serum-free medium and placed in the upper chamber, whereas 600μl 20% FBS-DMEM alone or containing 50 ng/ml SDF-1(Beyotime, Shanghai, CN) was added into the lower chamber. After incubation for 24h, non-invading cells were mechanically wiped using cotton swab, the inserts were fixed by 4% paraformaldehyde and stained with 0.5% Crystal violet solution (Muto Pure Chemical, Tokyo, Japan). The migrated cells were counted under light microscopy (×100 magnification).

To determine the effect of CXCR4 knockdown on apoptosis, cells of each subgroup were detected at 48 hours after transfection. For the analysis of CXCR4 expression on the response to PTX, at 24 hours after transfection, cells were treated with 20nM PTX for another 48 hours. Apoptosis analysis was performed using Annexin V-FITC/PI Apoptosis Detection Kit (YEASEN, Shanghai, CN) and cells were detected with flow cytometry on a FACScan (BD Biosciences, NJ, USA) in the corresponding procedures as described previously 21.

### Statistical analysis

We adopted Pearson χ2 test or Fisher's exact test to analyze the associations between CXCR4 expression and clinicopathological characteristics. Survival curves were plotted by the Kaplan-Meier method and compared using the log-rank test. Variables with P value <0.05 from univariate analysis were included into multivariate analysis. TNM stage was not included in the final multivariate analysis due to the association with lymph node metastasis. Multivariate analysis was performed by the Cox proportional hazards model. The statistical analyses were performed using SPSS 20.0 for Windows (SPSS Inc., Chicago, IL, USA). For* in vitro* studies, data were analyzed using GraphPad Prism software package (version 5; GraphPad Software Inc., La Jolla, CA, USA) and presented as the mean ± standard deviations (SD). Differences between two groups were analyzed using the Student's unpaired t-test. P values < 0.05 were considered statistically significant. Each experiment was repeated 3 times.

## Results

### Clinicopathological characteristics of ASC patients

**Figure 1:**
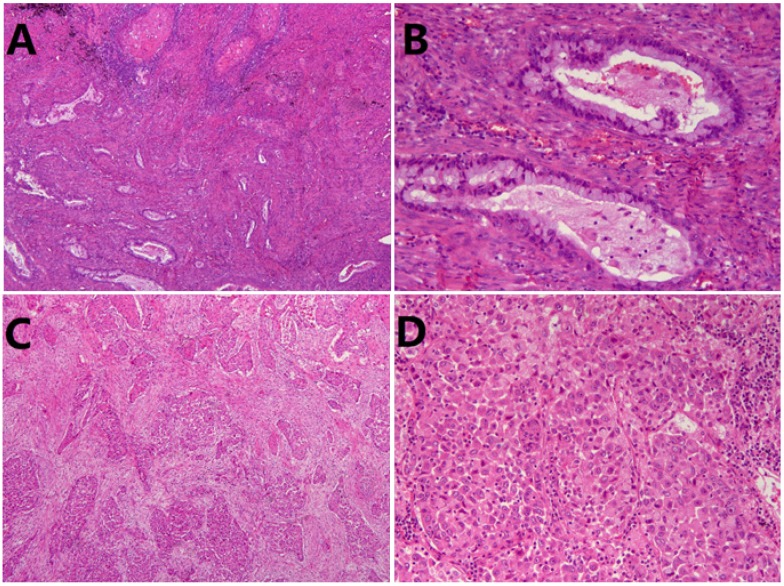
Representative images of subtype of lung adenosquamous carcinoma (ASC). (A) Non-solid ASC HE×50. (B) Non-solid ASC HE×200. (C) Solid ASC×50. (D) Solid ASC×200.

Seventy-eight patients were included in this study, consisting of 53 men (67.9%) and 25 women (32.1%), with a mean age of 63.4±9.2 years (range, 38-82 years). There were 49 (62.8%) smokers and 29 (37.2%) non-smokers. Histologically, 48(61.5%) were classified into non-solid ASC (Fig. 1A, B), while 30 (38.5%) individuals were classified into solid ASC (Fig. 1C, D). Pathologically, 37 (47.4%) patients demonstrated tumor lymph node metastasis, and 25 (32.1%) patients were diagnosed at stage I while 53 (67.9%) patients at advanced stage (II-III). Other clinicopathological characteristics were listed in Table 1.

### Correlations between CXCR4 expression and clinicopathological characteristics

**Figure 2:**
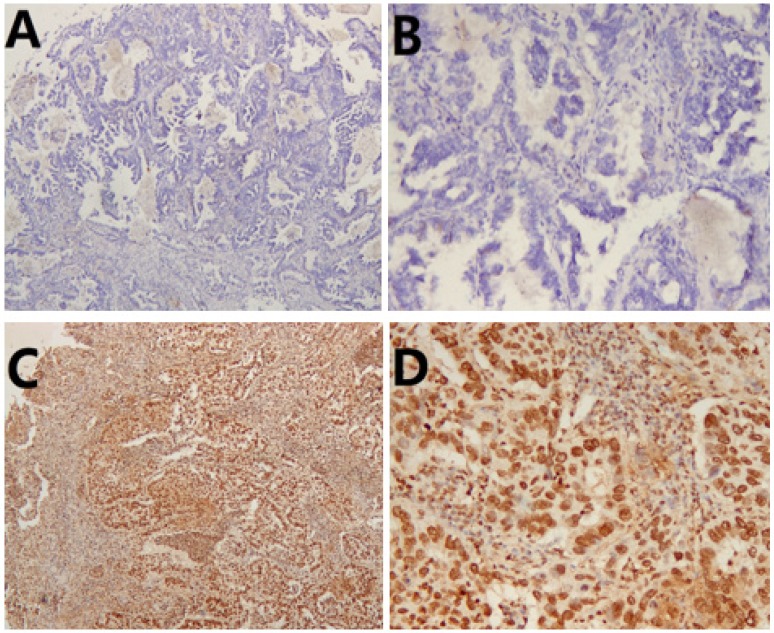
Immunohistochemical staining of CXCR4 in lung adenosquamous carcinoma (ASC). (A) Low CXCR4 staining ×50. (B) Low CXCR4 staining ×200. (C) High CXCR4 staining×50. (D) High CXCR4 staining×200.

To verify the relationship between CXCR4 expression and the clinicopathological features of lung ASC patients, we investigated the expression of CXCR4 in tumor tissues. As shown in Table 1, low and high expression of CXCR4 was observed in 33(42.3%) and 45 (57.7%) patients, respectively. High CXCR4 was significantly more frequently expressed in solid ASC (p=0.002). In addition, there was a positive correlation between high CXCR4 expression and lymph node metastasis (p=0.033). Meanwhile, high level of CXCR4 expression was significantly correlated with advanced TNM stage (II-III) (p=0.030). Representative images of immunohistochemical staining of CXCR4 were shown in Fig.2.

### Survival analysis

**Figure 3:**
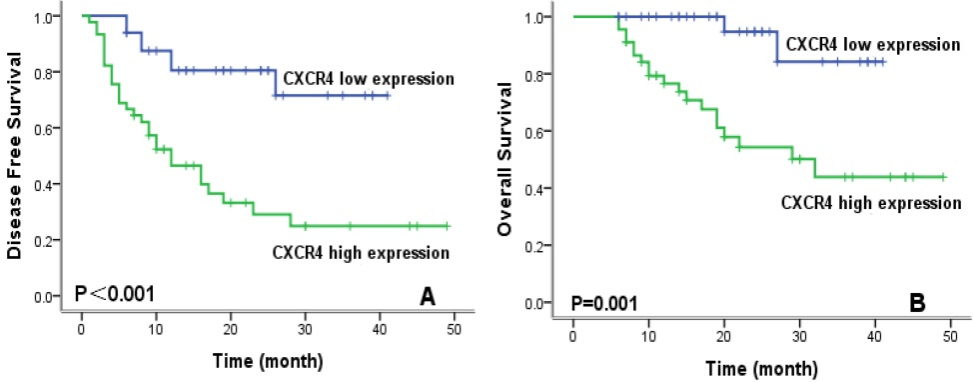
Kaplan-Meier survival curves for disease-free survival (A) and overall survival (B) according to CXCR4 expression.

**Figure 4:**
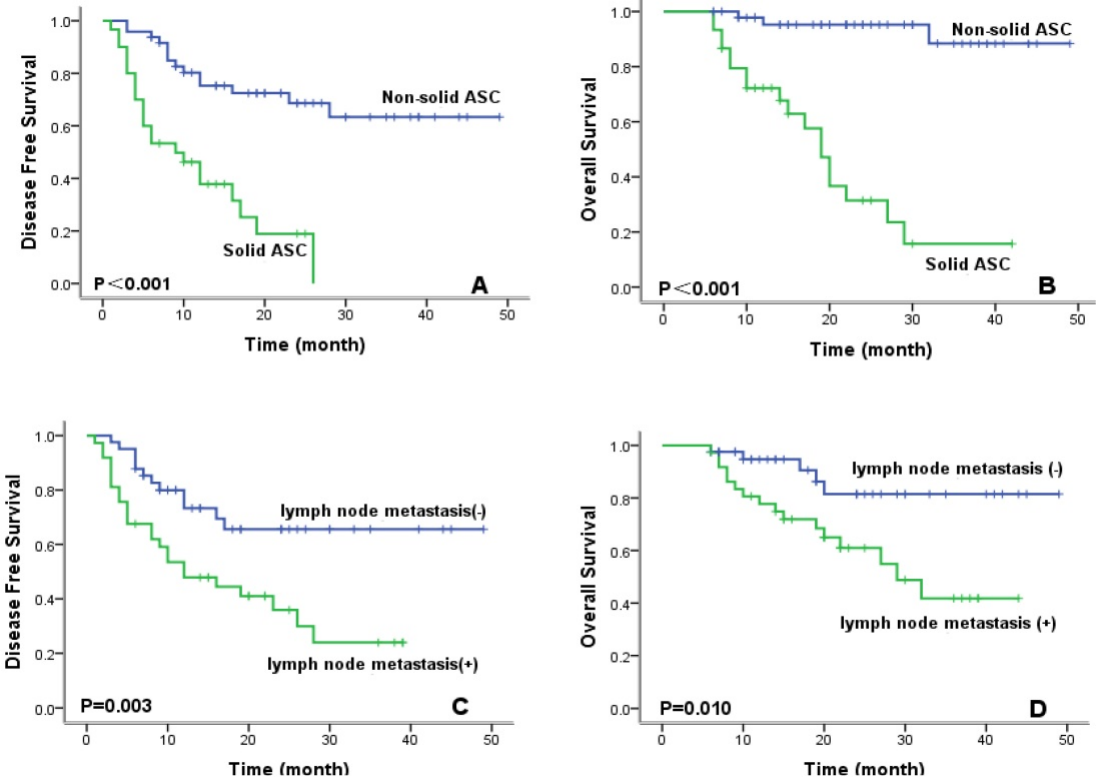
Kaplan-Meier survival curves for disease-free survival and overall survival according to ASC subtype (A, B) and lymph node metastasis (C, D).

To evaluate prognostic factors for survival in lung ASC patients, univariate and multivariate analyses were performed. In univariate analysis (Table 2), patients with high CXCR4 expression, solid ASC subtype, and lymph node metastasis exhibited significantly worse DFS and OS compared with their counterparts. Kaplan-Meier survival curves demonstrated that high CXCR4 expression (Fig.3), solid ASC and lymph node metastasis (Fig.4) indicated decreased DFS and OS. Moreover, poor tumor differentiation, and advanced TNM stage (II+III) were also significantly correlated with both worse DFS and OS. In addition, presence of micropapillary component in tumors indicated poorer DFS, while female patients and non-smokers had improved OS. In multivariate analysis, high CXCR4 expression and solid ASC were identified as independent prognostic factors of both poorer DFS and OS (Table 3, 4).

### High CXCR4 expression had no impact on ASC proliferation but enhanced invasiveness and chemoresistance* in vitro*

**Figure 5:**
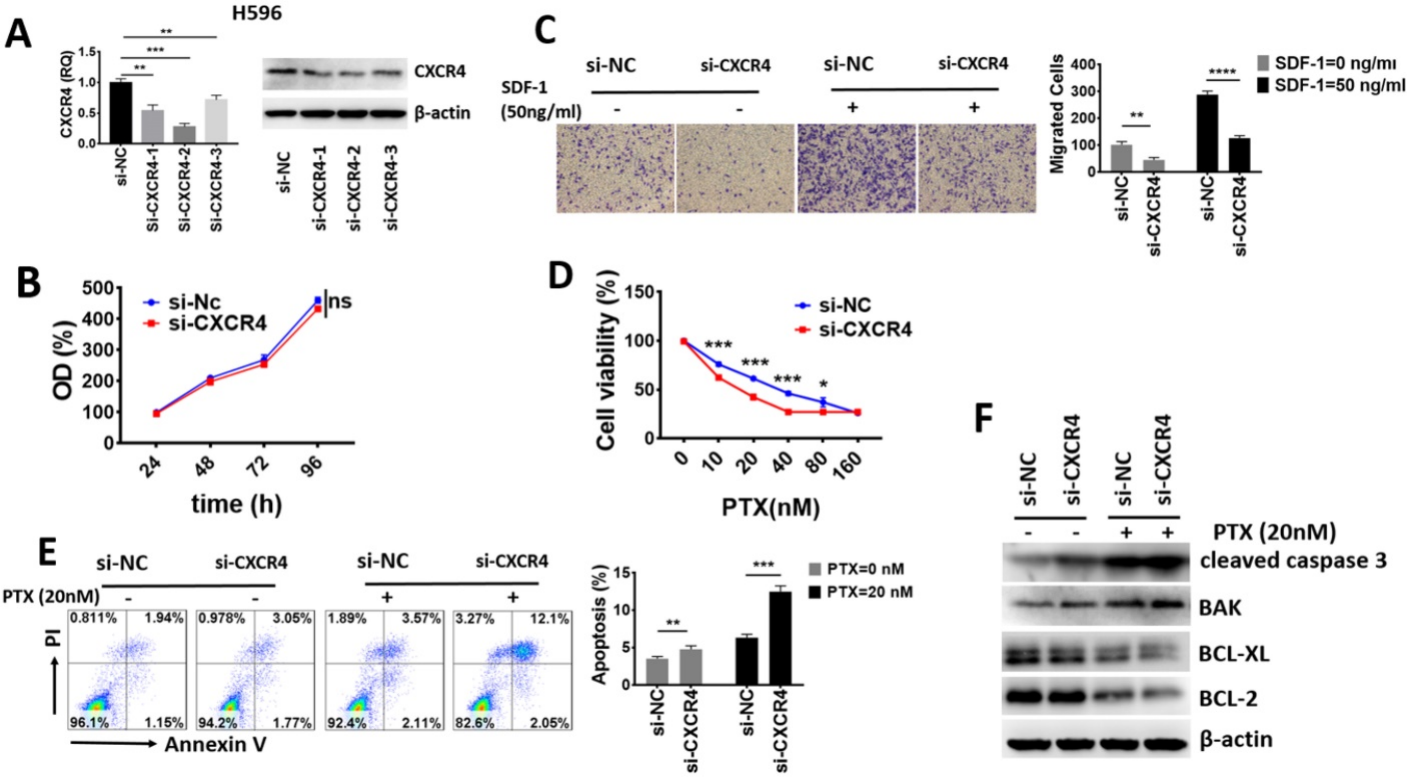
CXCR4 had no impact on proliferation but promoted cell migration, chemoresistance and inhibited apoptosis of lung ASC *in vitro*. (A) The effect of CXCR4 knockdown in H596 was validated through qRT-PCR and Western blot analyses. (B) The proliferative ability was assessed with CCK-8 assay at indicated times after transfection. (C)The migrative ability was assessed with Transwell assay. (D) Cell viability after increasing concentrations of PTX treatment for 72 h was assessed with CCK-8 assay. (E) Annexin V/ PI assays for detection of cell apoptosis after knockdown of CXCR4 and PTX treatment. (F) Western blot assays to analyze the apoptosis related protein expression after knockdown of CXCR4 and PTX treatment. β-actin was used as an internal reference. (ns: no significance, * p <0.05, **p <0.01, ***p <0.001).

To explore the biological function of CXCR4 in lung ASC, we selected human lung ASC cell line H596. We constructed 3 fragments of siRNA to knock down the expression of CXCR4, with Nc-CXCR4 being used as a control. The effect of CXCR4 silence was validated through qRT-PCR and Western blot. As shown in the Fig. 5A, all the 3 fragments of siRNA displayed great effect of knockdown. Since si-CXCR4-2 showed the greatest knockdown effect, we selected it for further study.

To study whether CXCR4 made a difference in tumor proliferation, CCK-8 assay was performed. As shown in Fig. 5B, CXCR4-silenced subgroup (si-CXCR4) demonstrated no significantly lower proliferative level than that of normal control (si-NC) group after transfection. Our results revealed that high CXCR4 level conferred no proliferation advantage in lung ASC.

To investigate the impact of CXCR4 on migration ability, we performed Transwell assays. The results in Fig. 5C revealed that high level of CXCR4 promoted the basal and SDF-1 directed cell migration in lung ASC.

We then examined whether CXCR4 influences the response to chemotherapeutic agents. As shown in Fig. 5D, CXCR4 downregulation significantly sensitized H596 cells to PTX. The IC50 value of si-NC and si-CXCR4 subgroup to PTX was 38.2 nM and 18.45 nM, respectively. These results demonstrated that high CXCR4 enhanced chemoresistance in lung ASC. We further determined cell apoptosis with flow cytometry assays and detected apoptosis-related proteins with Western blot assays. As shown in Fig. 5E, knock-down of CXCR4 significantly increased basal and PTX-mediated apoptosis in H596 cells compared with the control group. In addition, Western-blot assays from Fig. 5F indicated that pro-apoptotic proteins such as caspase 3 cleavage and BAK were significantly elevated while anti-apoptotic proteins including BCL-XL and BCL-2 were downregulated in basal and PTX-induced si-CXCR4 subgroup, suggesting that cells with high CXCR4 were more resistant to apoptosis. Taken together, our data proved that high CXCR4 expression had no impact on lung ASC proliferation, but played a crucial role in enhancing cell migration, chemoresistance and inhibiting apoptosis *in vitro*.

## Discussion

Adenosquamous carcinoma (ASC) accounts for 0.4% to 4.0% of lung cancer 4, 5, which is a rare histology type of NSCLC. Meanwhile, due to its highly aggressive biological characteristics, some potential patients with lung ASC had lost the opportunity for surgery before diagnosis. Therefore, it is difficult to collect a large sample size of resected lung ASC patients in a single institution. Our current study included 78 patients from which we examined the prognostic and clinicopathological features of resected lung ASC. Notably, we for the first time explored the clinical and potential biological role of CXCR4 in lung ASC.

Lung ASCs are morphologically mixed tumors containing adenomatous and squamous components. However, it remains unclear whether histological subtype of AC in ASC significantly influences prognosis. Zhu and his colleagues^22^ reported that patients with ASC containing acinar predominant AC had better survival than those with non-acinar predominant AC. More researchers reported no significant difference in RFS between patients with classical ASC and those showing solid ASC^23^. In our current work, we classified ASC into non-solid and solid subtypes according to predominant glandular component. Results from our data demonstrated that patients with solid ASC had significantly poorer prognosis, and that solid ASC was identified as an independent factor in predicting decreased DFS/OS in the multivariate analysis. To confirm the role of inherent mixed histological components in ASC tumors, multi-center studies with larger sample size and detailed subtyping of lung ASC are still needed.

PTX is one of the most applied first-line chemotherapeutic agents for NSCLC^24^. However, the efficacy of PTX might be gradually hampered by acquired resistance with the passage of time, which became a major adverse factor for patients' prognosis^25^. Therefore, identification of molecules that are involved in NSCLC relapse and prognosis might be of great significance to discover new biomarkers for early detection and therapeutic targets. Over the past few years, several molecular targeted drugs has been applied in the clinical treatment of NSCLC and achieved good effects, especially EGFR-TKI^26^,^27^. However, nearly all of these patients experience acquired resistance within 9-15 months, which strictly restricts its clinical use28. In our results, significant survival improvement for the efficacy of EGFR-TKI in management of lung ASC was not achieved (p=0.073). In this regard, novel specific molecular targets and alternative therapeutic interventions are urgently needed to manage the devastating disease.

Increasing evidence suggests that the CXCL12/CXCR4 chemokine axis plays an important role in migration and metastasis in various cancers^29^-^31^. In this study and for the first time, we examined the expression of CXCR4 and determined its correlation with clinicopathological characteristics in resected lung ASC. We found that 70 (89.7%) patient tumors expressed CXCR4, and that high level of CXCR4 was expressed in 45 (57.7%) cases. Moreover, high CXCR4 expression was significantly correlated with solid ASC, which demonstrated worse survival outcomes compared with non-solid ASC. In addition, high CXCR4 expression was positively correlated with lymph node metastasis and advanced TNM stages (II-III). These findings suggested that CXCR4 might be an important molecule closely associated with the invasion and metastasis of tumor, probably as one of the indices for determining the prognosis of lung ASC.

To further validate our findings, we explored the potential biological function of CXCR4 in lung ASC *in vitro*. We established our model by transfection using siRNA to silence CXCR4 expression. Firstly, we found that CXCR4 had no impact on proliferation capacity in CCK-8 assay, which could explain why CXCR4 expression had no significant correlation with tumor size clinically. However, CXCR4-silenced cells exhibited decreased basal and SDF-1 directed cell migration in transwell assay, which was parallel to our finding that CXCR4 was positively correlated with lymph node metastasis in clinic. Moreover, lung ASC cells with knockdown of CXCR4 exhibited lower IC50 value to paclitaxel and increased basal as well as PTX-mediated apoptosis, corresponding to decreased DFS/OS in tumors with high CXCR4 expression. Collectively, high CXCR4 might play a crucial role in promoting migration, enhancing chemoresistance and inhibiting apoptosis of lung ASC cells. Thus, we reasoned that CXCR4 might possess significant prognostic value and offer a novel therapeutic strategy to prevent or postpone the progression of lung ASC.

There are several limitations should be taken into consideration. First, this was a retrospective study and the inherent bias might limit the significance of clinical outcome. Meanwhile, the sample size included in our work was relatively small. Therefore, initiation of prospective studies, continuous collection of patients and clinical data are needed to further validate this issue in the future.

In summary, this study analyzed and provided the first evidence that CXCR4 was expressed in the majority of lung ASC tumor tissues. High CXCR4 expression promoted tumor invasiveness, enhanced chemoresistance and inhibited apoptosis of lung ASC *in vitro*. Clinically, High CXCR4 expression might be a novel marker in predicting a poor prognosis in patients with lung ASC and serve as a potential therapeutic target. In the future, in-depth explorations of the role of CXCR4 in promoting progression of lung ASC are warranted.

## Figures and Tables

**Table 1:** Correlation between CXCR4 expression and patient clinicopathological features

		CXCR4		
Variables	N (%)	low	high	P value
**Total**	78	33	45	
**Age (years)**				
mean ± SD	63.4±9.2			
range	38-82			
<60	29 (37.2)	10	19	0.282
≥60	49 (62.8)	23	26	
**Gender**				
male	53 (67.9)	21	32	0.485
female	25 (32.1)	12	13	
**Smoking history**				
No	29 (37.2)	13	16	0.729
Yes	49 (62.8)	20	29	
**Proportion of ASC component**				
SCC predominant	32 (41.0)	10	22	0.213
Balanced	18 (23.1)	10	8	
AC predominant	28 (35.9)	13	15	
**Subtype of ASC**				
non-solid	48 (61.5)	27	21	**0.002**
solid	30 (38.5)	6	24	
**Micropapillary**				
absent	46 (59.0)	21	25	0.473
present	32 (41.0)	12	20	
**Differentiation**				
moderate	25 (32.1)	14	11	0.093
poor	53 (67.9)	19	34	
**Pleural invasion**				
absent	38 (48.7)	17	21	0.672
present	40 (51.3)	16	24	
**Lymphovascular invasion**				
absent	60 (77.0)	22	38	0.066
present	18 (23.0)	11	7	
**Tumor size**				
≤3cm	30 (38.5)	14	16	0.538
>3cm	48 (61.5)	19	29	
**T stage**				
T1	17 (21.8)	8	9	0.391
T2	43 (55.1)	16	27	
T3	16 (20.5)	9	7	
T4	2 (2.6)	0	2	
**Lymph node metastasis**				
absent	41 (52.6)	22	19	**0.033**
present	37 (47.4)	11	26	
**TNM stage**				
I	25 (32.1)	15	10	**0.030**
II-III	53 (67.9)	18	35	
**EGFR status**				
wild type	51 (65.4)	23	28	0.493
mutated	27 (34.6)	10	17	

^a^ Bold values indicate statistical significance (*p* < 0.05). Abbreviation: SD, standard deviation; ASC, adenosquamous carcinoma; SCC,squamous cell carcinoma; AC, adenocarcinoma.

**Table 2:** Univariate analysis of patient's survival

	DFS				OS				
Variables	Mean (month)	95% CI	P value		Mean (month)		95% CI		P value
**Age (years)**									
<60	27.1	19.1-35.0	0.837		39.9		33.7-46.1		0.211
≥60	23.8	19.2-28.4			29.6		25.5-33.8		
**Gender**									
male	26.8	20.5-33.3	0.453		31.5		25.4-37.6		**0.003**
female	28.2	21.3-35.0			41.5		37.9-45.2		
**Smoking history**									
No	28.0	19.9-32.9	0.872		41.7		38.3-45.2		**0.001**
Yes	26.4	21.3-34.7			30.2		23.9-36.6		
**Proportion of ASC component**									
SCC predominant	30.4	22.6-38.2	0.955		34.3		27.0-41.6		0.149
Balanced	22.6	15.0-30.2			29.5		22.6-36.4		
AC predominant	24.6	18.3-31.0			37.6		32.2-43.0		
**Subtype of ASC**									
non-solid	35.8	30.1-41.5	**<0.001**		46.0		42.7-49.3		**<0.001**
solid	11.4	8.2-15.0			20.7		15.8-25.5		
**Micropapillary**									
absent	32.3	25.8-38.7	**0.041**		32.1		27.1-37.1		0.933
present	19.8	14.2-25.5			36.8		30.9-42.7		
**Differentiation**									
moderate	38.6	31.4-45.8	**0.003**		45.6		41.1-50.1		**0.004**
poor	18.2	14.0-22.3			28.3		23.8-32.8		
**Pleural invasion**									
absent	28.1	20.8-35.4	0.782		36.3		30.1-42.4		0.860
present	25.1	19.2-31.1			34.2		29.1-39.3		
Lymphovascular invasion									
absent	28.6	23.0-34.2	0.705		35.7		30.7-40.8		0.599
present	21.9	14.6-29.3			34.4		27.8-40.9		
Tumor size									
≤3cm	22.0	15.8-28.2	0.600		33.6		27.9-38.5		0.630
>3cm	29.4	23.2-35.6			36.1		30.6-41.6		
T stage									
T1	21.0	15.9-26.2	0.833		29.6		22.0-37.3		0.130
T2	27.5	21.0-34.0			39.0		33.6-44.3		
T3	23.6	15.7-31.4			28.7		22.0-35.4		
T4	7.0	1.5-12.5			8.5		5.0-12.0		
**Lymph node metastasis**									
absent	35.4	29.1-41.8	**0.003**		42.8		37.9-47.8		**0.010**
present	18.0	13.1-22.8			29.0		23.7-34.3		
TNM stage									
I	38.4	31.1-45.8	**0.009**		45.2		40.3-50.2		**0.015**
II-III	19.8	15.6-24.1			30.5		25.9-35.0		
EGFR status									
wild type	26.6	20.9-32.3	0.958		31.7		26.6-36.8		0.073
mutated	27.4	19.7-35.0			41.2		35.3-47.1		
CXCR4 expression									
low	33.4	28.4-38.3	**<0.001**		38.4		35.1-41.8		**<0.001**
high	19.7	13.9-25.6			30.7		24.9-36.6		
									

^a^ Bold values indicate statistical significance (*p* < 0.05). Abbreviation: DFS, disease free survival; OS, overall survival; CI, confidence interval; ASC, adenosquamous carcinoma; SCC,squamous cell carcinoma; AC, adenocarcinoma.

**Table 3:** Multivariate analysis of patient's DFS

	DFS		
Variables	HR	95% CI	P value
Subtype of ASC (non-solid vs. solid)	0.242	0.109-0.538	**0.001**
Micropapillary (present vs. absent)	1.931	0.247-1.084	0.081
Differentiation (moderate vs. poor)	0.737	0.271-2.004	0.550
Lymph node metastasis (absent vs. present)	0.557	0.265-1.174	0.124
CXCR4 expression (low vs. high)	0.352	0.147-0.845	**0.019**

^a^ Bold values indicate statistical significance (*p* < 0.05). Abbreviation: DFS, disease free survival; HR, hazard ratio; CI, confidence interval; ASC, adenosquamous carcinoma.

**Table 4:** Multivariate analysis of patient's OS

	OS		
Variables	HR	95% CI	P value
Gender (male vs. female)	2.248	0.524-9.642	0.276
Smoking history (no vs. yes )	0.232	0.049-1.099	0.066
Subtype of ASC (non-solid vs. solid)	0.151	0.034-0.665	**0.012**
Differentiation (moderate vs. poor)	0.542	0.104-2.836	0.469
Lymph node metastasis (absent vs. present)	0.404	0.135-1.206	0.104
CXCR4 expression (low vs. high)	0.188	0.040-0.879	**0.034**

^a^ Bold values indicate statistical significance (*p* < 0.05). Abbreviation: OS, overall survival; HR, hazard ratio; CI, confidence interval; ASC, adenosquamous carcinoma.

## Abbreviations

AC: adenocarcinoma
ASC: adenosquamous carcinoma
CI: confidence interval
CXCR4: C-X-C chemokine receptor 4
SDF-1: stromal cell-derived factor 1
DFS: disease free survival
EGFR: epidermal growth factor receptor
EGFR-TKI: epidermal growth factor receptor tyrosine kinase inhibitor
HR: hazard ratio
OS: overall survival
SCC: squamous cell carcinoma
SD: standard deviation
TNM: tumor-node-metastasis
PTX: paclitaxel

## References

[1] Alberg AJ, Brock MV, Samet JM (2005). Epidemiology of lung cancer: looking to the future. J Clin Oncol.

[2] Cooke DT, Nguyen DV, Yang Y (2010). Survival comparison of adenosquamous, squamous cell, and adenocarcinoma of the lung after lobectomy. Ann Thorac Surg.

[3] Travis WD, Brambilla E, Nicholson AG (2015). The 2015 World Health Organization Classification of Lung Tumors: Impact of Genetic, Clinical and Radiologic Advances Since the 2004 Classification. J Thorac Oncol.

[4] Gawrychowski J, Brulinski K, Malinowski E, Papla B (2005). Prognosis and survival after radical resection of primary adenosquamous lung carcinoma. Eur J of cardiothorac Surg.

[5] Nakagawa K, Yasumitu T, Fukuhara K, Shiono H, Kikui M (2003). Poor prognosis after lung resection for patients with adenosquamous carcinoma of the lung. Ann Thorac Surg.

[6] Smith MC, Luker KE, Garbow JR (2004). CXCR4 regulates growth of both primary and metastatic breast cancer. Cancer Res.

[7] Jiang YP, Wu XH, Shi B, Wu WX, Yin GR (2006). Expression of chemokine CXCL12 and its receptor CXCR4 in human epithelial ovarian cancer: an independent prognostic factor for tumor progression. Gynecol Oncol.

[8] Zeelenberg IS, Ruuls-Van Stalle L, Roos E (2003). The chemokine receptor CXCR4 is required for outgrowth of colon carcinoma micrometastases. Cancer Res.

[9] Mori T, Doi R, Koizumi M (2004). CXCR4 antagonist inhibits stromal cell-derived factor 1-induced migration and invasion of human pancreatic cancer. Mol Cancer Ther.

[10] Su L, Zhang J, Xu H (2005). Differential expression of CXCR4 is associated with the metastatic potential of human non-small cell lung cancer cells. Clin Cancer Res: an official journal of the American Association for Cancer Research.

[11] Phillips RJ, Mestas J, Gharaee-Kermani M (2005). Epidermal growth factor and hypoxia-induced expression of CXC chemokine receptor 4 on non-small cell lung cancer cells is regulated by the phosphatidylinositol 3-kinase/PTEN/AKT/mammalian target of rapamycin signaling pathway and activation of hypoxia inducible factor-1alpha. J Biol Chem.

[12] Minamiya Y, Saito H, Takahashi N (2010). Expression of the chemokine receptor CXCR4 correlates with a favorable prognosis in patients with adenocarcinoma of the lung. Lung Cancer.

[13] Spano JP, Andre F, Morat L (2004). Chemokine receptor CXCR4 and early-stage non-small cell lung cancer: pattern of expression and correlation with outcome. Ann Oncol.

[14] Wagner PL, Hyjek E, Vazquez MF (2009). CXCL12 and CXCR4 in adenocarcinoma of the lung: association with metastasis and survival. J Thorac Cardiovasc Surg.

[15] Travis WD, Brambilla E, Noguchi M (2011). International association for the study of lung cancer/american thoracic society/european respiratory society international multidisciplinary classification of lung adenocarcinoma. J Thorac Oncol.

[16] Lu CL, Guo J, Gu J (2014). CXCR4 heterogeneous expression in esophageal squamous cell cancer and stronger metastatic potential with CXCR4-positive cancer cells. Dis Esophagus.

[17] Chao L, Yi-Sheng H, Yu C (2014). Relevance of EGFR mutation with micropapillary pattern according to the novel IASLC/ATS/ERS lung adenocarcinoma classification and correlation with prognosis in Chinese patients. Lung Cancer.

[18] Goldstraw P, Crowley J, Chansky K (2007). The IASLC Lung Cancer Staging Project: proposals for the revision of the TNM stage groupings in the forthcoming (seventh) edition of the TNM Classification of malignant tumours. J Thorac Oncol.

[19] Gu J, Ding JY, Lu CL (2013). Overexpression of CD88 predicts poor prognosis in non-small-cell lung cancer. Lung Cancer.

[20] Zhao GY, Lin ZW, Lu CL (2015). USP7 overexpression predicts a poor prognosis in lung squamous cell carcinoma and large cell carcinoma. Tumour Biol.

[21] Xu F, Gu J, Lu C (2019). Calpain-2 Enhances Non-Small Cell Lung Cancer Progression and Chemoresistance to Paclitaxel via EGFR-pAKT Pathway. Int J Biol Sci.

[22] Zhu L, Jiang L, Yang J, Gu W, He J (2018). Clinical characteristics and prognosis of patients with lung adenosquamous carcinoma after surgical resection: results from two institutes. J Thorac Dis.

[23] Wang R, Pan Y, Li C (2014). Analysis of major known driver mutations and prognosis in resected adenosquamous lung carcinomas. J Thorac Oncol.

[24] Ettinger DS, Wood DE, Aisner DL (2017). Non-Small Cell Lung Cancer, Version 5.2017, NCCN Clinical Practice Guidelines in Oncology. J Natl Compr Canc Ne.

[25] Liu R, Liu X, Zheng Y (2014). MicroRNA-7 sensitizes non-small cell lung cancer cells to paclitaxel. Oncol Lett.

[26] Maemondo M, Inoue A, Kobayashi K (2010). Gefitinib or chemotherapy for non-small-cell lung cancer with mutated EGFR. N Engl J Med.

[27] Mitsudomi T, Morita S, Yatabe Y (2010). Gefitinib versus cisplatin plus docetaxel in patients with non-small-cell lung cancer harbouring mutations of the epidermal growth factor receptor (WJTOG3405): an open label, randomised phase 3 trial. Lancet Oncol.

[28] Mok TS, Wu YL, Thongprasert S (2009). Gefitinib or carboplatin-paclitaxel in pulmonary adenocarcinoma. N Engl J Med.

[29] Kryczek I, Wei S, Keller E, Liu R, Zou W (2007). Stroma-derived factor (SDF-1/CXCL12) and human tumor pathogenesis. Am J Physiol Cell Physiol.

[30] Lu C, Xu F, Gu J (2015). Clinical and biological significance of stem-like CD133(+)CXCR4(+) cells in esophageal squamous cell carcinoma. J Thorac Cardiovasc Surg.

[31] Lu CL, Ji Y, Ge D, Guo J, Ding JY (2011). The expression of CXCR4 and its relationship with matrix metalloproteinase-9/vascular endothelial growth factor in esophageal squamous cell cancer. Dis Esophagus.

